# Video-Oculography-Assisted Head Impulse Test and Caloric Testing for Detecting Stroke in Acute Vertigo Patients via Modified HINTS Plus

**DOI:** 10.3390/jcm10194471

**Published:** 2021-09-28

**Authors:** Timo Siepmann, Cosima Gruener, Erik Simon, Annahita Sedghi, Hagen H. Kitzler, Lars-Peder Pallesen, Jessica Barlinn, Heinz Reichmann, Volker Puetz, Kristian Barlinn

**Affiliations:** 1 Department of Neurology, University Hospital Carl Gustav Carus, Technische Universität Dresden, 01307 Dresden, Germany; cosima.gruener@ukdd.de (C.G.); erik.simon@ukdd.de (E.S.); annahita.sedghi@ukdd.de (A.S.); lars-peder.pallesen@ukdd.de (L.-P.P.); jessica.barlinn@ukdd.de (J.B.); heinz.reichmann@ukdd.de (H.R.); volker.puetz@ukdd.de (V.P.); kristian.barlinn@ukdd.de (K.B.); 2 Institute of Diagnostic and Interventional Neuroradiology, University Hospital Carl Gustav Carus, Technische Universität Dresden, 01307 Dresden, Germany; hagen.kitzler@ukdd.de

**Keywords:** stroke, vertigo, acute vestibular syndrome, HINTS, V-HIT, video-oculography, dizziness, neurology

## Abstract

Background: We assessed whether detection of stroke underlying acute vertigo using HINTS plus (head-impulse test, nystagmus type, test of skew, hearing loss) can be improved by video-oculography for automated head-impulse test (V-HIT) analysis. Methods: We evaluated patients with acute vestibular syndrome (AVS) presenting to the emergency room using HINTS plus and V-HIT-assisted HINTS plus in a randomized sequence followed by cranial MRI and caloric testing. Image-confirmed posterior circulation stroke or vertebrobasilar TIA were the reference standards to calculate diagnostic accuracy. We repeated statistical analysis for a third protocol that was composed post hoc by replacing the head-impulse test with caloric testing in the HINTS plus protocol. Results: We included 30 AVS patients (ages 55.4 ± 17.2 years, 14 females). Of these, 11 (36.7%) had posterior circulation stroke (*n* = 4) or TIA (*n* = 7). Acute V-HIT-assisted HINTS plus was feasible and displayed tendentially higher accuracy than conventional HINTS plus (sensitivity: 81.8%, 95% CI 48.2–97.7%; specificity 31.6%, 95% CI 12.6–56.6% vs. sensitivity 72.7%, 95% CI 39.0–94.0%; specificity 36.8%, 95% CI 16.3–61.6%). The new caloric-supported algorithm showed high accuracy (sensitivity 100%, 95% CI 66.4–100%; specificity 66.7%, 95% CI 41–86.7%). Conclusions: Our study provides pilot data on V-HIT-assisted HINTS plus for acute AVS assessment and indicates the diagnostic value of integrated acute caloric testing.

## 1. Introduction

Dizziness accounts for up to 4–6% of all emergency department visits, and 4% of these patients have an underlying cerebrovascular pathology [1]. Although vertebrobasilar stroke frequently presents with sole acute vestibular syndrome (AVS), defined as new and persistent acute vertigo or dizziness, with additional nausea/vomiting, head motion intolerance, or new gait unsteadiness, its detection is missed in up to one third of these patients [2]. However, vestibular neuritis is by far the most common cause of AVS, and therefore, rapid diagnostic discrimination is crucial to initiate acute stroke care in those with AVS of cerebrovascular origin [3]. As immediate cranial magnetic resonance imaging (MRI) is frequently not available, several tools have been designed to detect stroke patients among those presenting with AVS at the emergency department including HINTS plus (head-impulse test [HIT], nystagmus type, test of skew, hearing loss) and video-oculography-assisted HIT (V-HIT) [3,4,5]. 

We aimed to explore the diagnostic performance of V-HIT-assisted HINTS plus in the identification of acute stroke in patients presenting with AVS.

## 2. Materials and Methods

### 2.1. Participants and Protocol

We conducted a diagnostic randomized open-label method comparison pilot study at the emergency department of a tertiary stroke center in Germany (University Hospital Carl Gustav Carus, Dresden). We included consecutive patients with AVS presenting to our emergency department within 7 days after onset of symptoms. We excluded patients with postural vertigo defined as repeated, brief periods of spinning sensations following changes in the position of the head associated by nausea with positive Dix–Hallpike or roll test, visual impairment with inability of gaze stabilization, cervical spine impairment compromising conduction of head impulse test, history of psychogenic vertigo, consumption of alcohol or drugs, known ocular or vestibular disorder, or any other condition resulting in inability to participate in vertigo assessment. 

Following medical history and neurological physical examination, all study participants underwent conventional HINTS plus (head-impulse test [HIT], nystagmus type, test of skew, hearing loss) and V-HIT-assisted HINTS plus in a random derangement of test order. An investigator (K.B.) generated the allocation sequence using a web-based random number generator. Additionally, we performed a detailed diagnostic workup including caloric testing to detect peripheral vestibular dysfunction and cranial MRI to detect ischemic lesions in the posterior circulation. Evaluation of cMRI was performed after index vertigo assessments (HINTS plus and V-HIT-assisted HINTS plus) by experienced neuroradiologists blinded to study specific procedures. When clinical but not imaging assessment led to suspicion of stroke, MRI was repeated after 48–72 h from study inclusion. Imaging-confirmed posterior circulation stroke or vertebrobasilar TIA as defined by the Guidelines for the Prevention of Stroke in Patients with Stroke and Transient Ischemic Attack of the American Heart Association Stroke Council and with an ABCD2 score ≥ 4 points and/or other focal neurological signs were defined as reference standard to calculate diagnostic performance of applied vertigo assessment protocols [6,7].

### 2.2. HINTS Plus 

A stroke fellow (C.T.) performed the HINTS plus test as consecutive performance of HIT, assessment of nystagmus characteristics, test of skew via cover-uncover test, and bilateral hearing assessment by finger rubbing as previously described [3].

### 2.3. V-HIT-Assisted HINTS Plus

Video-oculography was performed using a high-speed compact camera (220 Hz sampling rate) attached to lightweight goggles (EyeSeeCam HIT, Interacoustics, Middelfart, Denmark) for quantitative recording of eye and head movements during head-impulse test as previously described [5,8]. Briefly, in a sitting position 1.5 m in front of a fixation target at eye level, the camera system captures usually undetectable covert saccades during the head movement as well as overt saccades that occur after the head movement and are usually detectable clinically.

### 2.4. Caloric Testing

Warm and cold-water stimulation caloric testing was performed separately for each ear with video-oculography-assisted detection of vestibular nystagmus, with absent or decreased response indicating peripheral vestibular dysfunction as previously described [9]. 

### 2.5. Cranial Magnetic Resonance Imaging 

A standardized cranial stroke imaging protocol was used to provide emergency imaging diagnostics to detect posterior fossa infarction: T2 turbo spin echo (axial and sagittal acquisition; slice thickness 3 mm), epiplanar diffusion imaging with b values 0 and 1000 (axial acquisition; slice thickness 3 mm), apparent diffusion coefficient (ADC) mapping, and T2 gradient echo (GRE) sequence (axial acquisition; slice thickness 3 mm). A 3.0 Tesla Siemens Magnetom Verio MRI Scanner (Siemens Healthineers, Erlangen, Germany) was used. Acute ischemia was determined by a focal lesion diffusion restriction caused by cytotoxic edema, with cell swelling and extracellular space reduction confirmed by a lesion ADC decrease. In addition, GRE differentiated underlying hemorrhage.

### 2.6. Caloric Testing Assisted NTS Plus

While the initial main goal of this study was to determine the diagnostic value of adding video-oculography to the HINTS plus test algorithm, our preliminary observations of limited accuracy for both protocols led us to compose and evaluate a new testing protocol post hoc by replacing HIT results with the results of caloric testing in the statistical analysis of accuracy of the HINTS protocol. Thus, our suggested new testing algorithm combines caloric testing with test of nystagmus, skew, and hearing (Cal-NTS plus).

### 2.7. Statistical Analysis

Analyses were performed using STATA (Version 12.1, StataCorp., College Station, TX, USA). Given the exploratory nature of the pilot study, we did not conduct a sample size calculation. Data are reported as mean ± standard deviation, median (interquartile range, IQR), or percentage according to type and distribution. Between-group comparisons were conducted using chi-squared test, Fisher’s exact test, Student’s *t*-test, and Wilcoxon rank-sum test, where appropriate. Diagnostic performance parameters including sensitivity, specificity, positive and negative predictive value (PPV and NPV), overall accuracy, and 95% confidence intervals (95% CI) were computed for C-HINTS plus, V-HIT-assisted-HINTS plus, and Cal-NTS plus. The equality of sensitivity and specificity for these approaches was tested using the McNemar’s chi-squared test. For this purpose, sensitivities and specificities were compared separately among diseased and non-diseased patients. *p*-value ≤ 0.05 was considered statistically significant. 

## 3. Results

**Figure 1:**
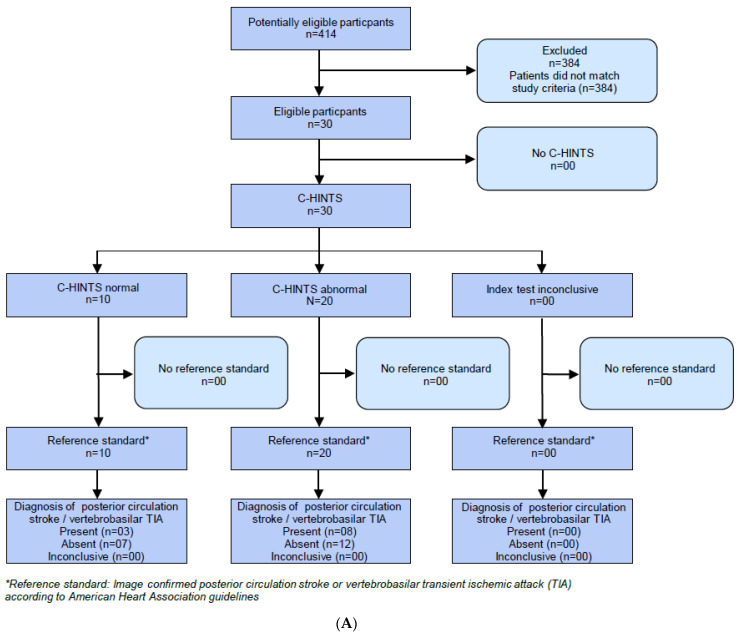

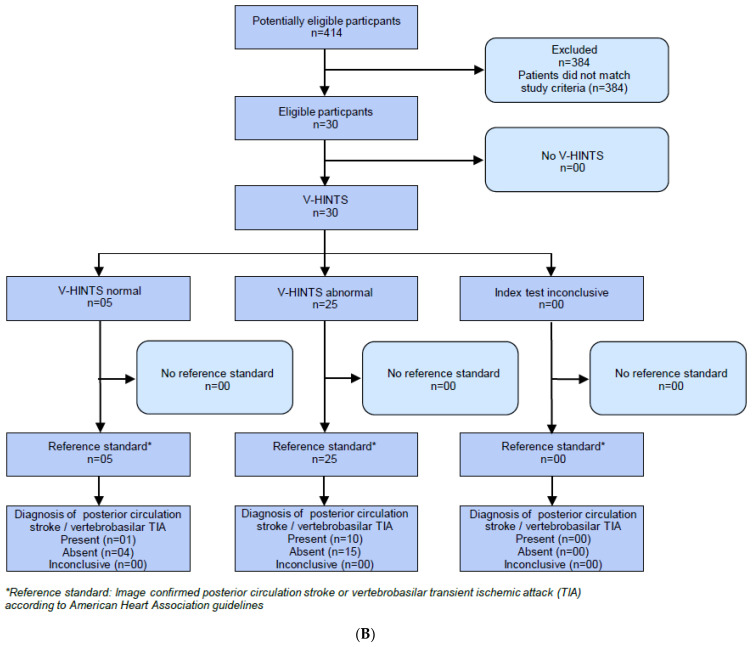

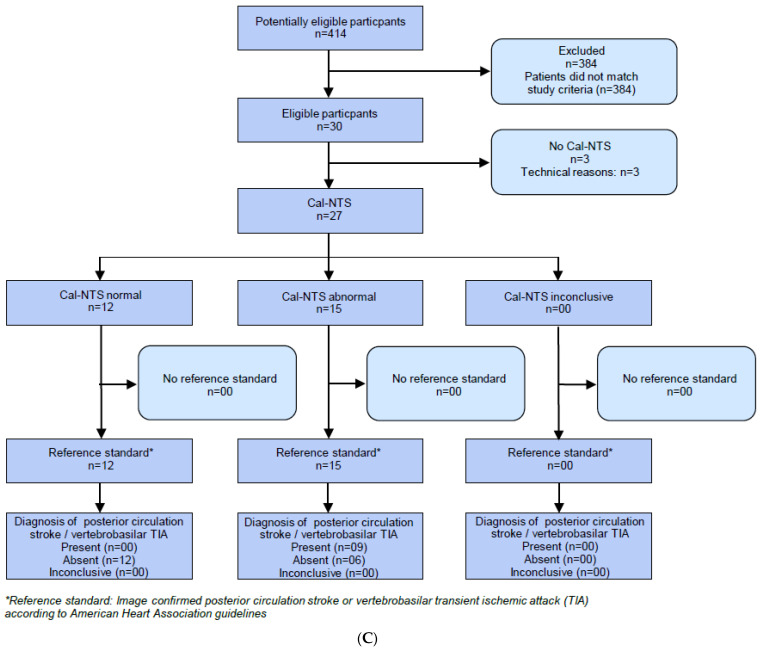
STARD Flow diagram. (**A**) C-HINTS plus; (**B**) V-HIT assisted HINTS plus; (**C**) Cal-NTS plus.

During the 12-month study period, we included *n* = 30 AVS patients (age 55.4 ± 17.2 years, F/M 0.88), and of these, *n* = 11 (36.7%) displayed an underlying cerebrovascular pathology with MRI-confirmed posterior circulation ischemic stroke (*n* = 4) or vertebrobasilar TIA with an ABCD2 score ≥ 4 (*n* = 7). The median elapsed time between symptom onset and cMRI was 1 day (IQR, 2). The STARD flow diagram is shown in Figure 1. 

No adverse events were noted from any of the performed vertigo assessments. We noted no indeterminate index test or reference standard results except for missing caloric tests from three patients, which were handled as a complete case analysis.

**Figure 2:**
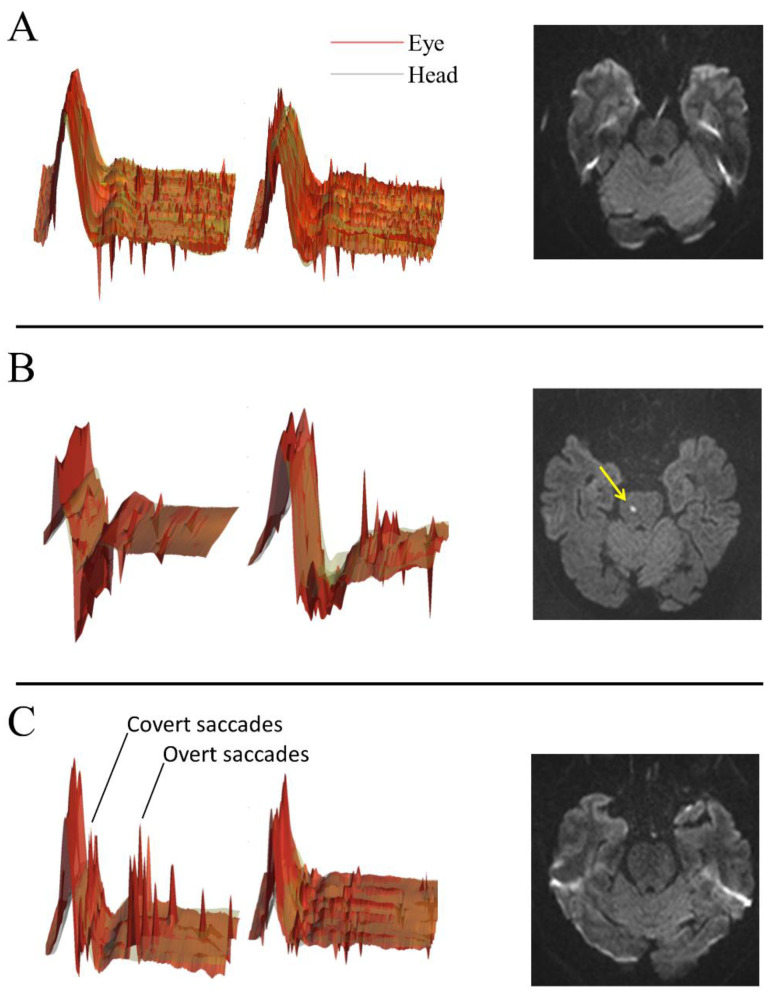
Video-oculography and cranial MRI correlates. (**A**) Conventional and V-HIT-assisted HINTS plus with an abnormal pattern, but normal caloric testing and brainstem and cerebellar MRI without pathological findings (diffusion trace map). (**B**) Abnormal pattern on conventional and V-HIT-assisted HINTS plus suggestive of a central lesion. Right paramedian punctuate diffusion restriction of an acute pontine infarction (yellow arrow). (**C**) Benign pattern on both HINTS plus examinations suggestive of peripheral vestibulopathy, unremarkable diffusion MRI, caloric testing confirmed unilateral vestibulopathy on the right side.

V-HIT-assisted HINTS plus tended to perform slightly better than C-HINTS plus (sensitivity 81.8%, 95% CI 48.2–97.7%; specificity 31.6%, 95% CI 12.6–56.6% vs. sensitivity 72.7%, 95% CI 39.0–94.0%; specificity 36.8%, 95% CI 16.3–61.6%). Replacing the head impulse test with caloric testing in the HINTS protocol seemed to further improve the diagnostic performance of the test with respect to the detection of a cerebrovascular cause of AVS (sensitivity 100%, 95% CI 66.4–100%; specificity 66.7%, 95% CI 41–86.7%). The sensitivities and specificities for the detection of the cerebrovascular cause of AVS did not differ (*p* > 0.05) among the three approaches. However, only the caloric testing-based algorithm was more frequently suggestive of central pathology in the group of patients with underlying stroke or TIA than in those without (*p* = 0.001, Table 1) The diagnostic accuracy parameters are detailed in Table 2. Exemplary findings from the V-HIT and MRI assessment are shown in Figure 2. 

## 4. Discussion

The major finding of this study is that V-HIT-assisted HINTS plus is a feasible tool to screen patients with AVS for underlying stroke with slightly increased diagnostic performance when compared to the conventional HINTS plus testing algorithm. Moreover, our study provides pilot data for testing the hypothesis that replacing the head-impulse test with rapid caloric testing leads to further improvement of the diagnostic accuracy of HINTS plus.

The HINTS examination has revolutionized the acute assessment of patients with AVS, and initial research has even suggested that the clinical algorithm outperforms cranial MRI in the diagnostic discrimination of peripheral and cerebrovascular etiology within the first 2 days after symptom onset [4]. However, a recent meta-analysis synthesizing data from 617 AVS patients found that HINTS in isolation cannot rule out central causes when performed by emergency physicians because of limited sensitivity (83%) and specificity (44%) [10]. In fact, test sensitivity and specificity seem to be highly dependent on the degree of the training of physicians, and clinicians with less experience are more likely to miss positive HITs [11,12,13]. Moreover, the initial misclassification of the type of vertigo may lead to a diagnostic error because the test algorithm lacks precision when it is not applied to the appropriate clinical scenario [14]. It is noteworthy that the most frequent misclassification of vertigo is a consequence of the failure to capture spontaneous nystagmus suppressed by visual fixation, resulting in a diagnosis of positional vertigo. Viewed in conjunction with our study, it may be concluded that the wide use of HINTS plus benefits from the supplementation of objective diagnostic technology to balance heterogeneity in the degree of training among physicians applying the test. 

Consistent with previous research, we observed a tendentially increased diagnostic accuracy in detecting cerebrovascular causes of AVS when video-oculography was performed to detect eye movements in the head-impulse test that are not visible to the human eye [8]. It is noteworthy that the confidence intervals for sensitivity were overlapping, possibly limiting the interpretability of this increase. Moreover, the observed slight increase in diagnostic accuracy needs to be balanced with the increase in technical demands by adding video-oculography to the testing protocol, even though our data support the feasibility of the technique as part of the emergency work up. Because of the limited improvements achieved by using video-oculography as an integrative part of standardized vertigo assessment, we went on and composed a new testing protocol post hoc by combining caloric testing with nystagmus assessment, test of skew, and test of hearing. Repeated statistical analysis revealed a further increase in diagnostic performance for the new protocol, resulting in a rate of false negative results of zero. However, caloric testing was not part of the investigational diagnostic test algorithms, as it was performed after the emergency work up. Therefore, our results need to be interpreted with caution and we cannot comment on the feasibility of caloric-testing-assisted vertigo assessment at initial presentation to the emergency room. However, modified caloric testing protocols were shown to provide a feasible procedure for swift bedside evaluation of vestibular function outside the vestibular laboratory [15]. Viewed in conjunction with our data, these advances form a basis for follow-up research in a larger sample of AVS to determine the feasibility and diagnostic accuracy of rapid caloric testing as part of the proposed Cal-NTS plus algorithm to detect underlying stroke. However, it is noteworthy that technical improvements of the HINTS plus test might only increase diagnostic performance in clinical practice if the selection of patients to receive the assessment is done adequately. In fact, a recent retrospective chart review study in a large cohort of patients with acute vertigo (*n* = 2309) revealed the limited diagnostic value of HINTS plus when performed by emergency room physicians, most likely attributable to the improper evaluation of clinical criteria to receive the HINTS plus exam [16]. The relatively long period of a maximum of 7 days between the onset of vertigo and inclusion may have affected diagnostic performance in our study population. A narrower time window might be useful in follow-up research to facilitate diagnostic discrimination. However, this limitation applies equally to all investigative testing algorithms. Thus, it is unlikely that comparability between assessments has been compromised by the onset-to-inclusion time period. 

## 5. Conclusions

While video-oculography for the analysis of HIT may slightly improve the diagnostic performance of the HINTS assessment, a combination of caloric testing, nystagmus assessment, test of skew, and test of hearing might be more effective in detecting stroke in AVS patients. Although the generalizability of our observations requires confirmation in a large sample of AVS patients, the randomized testing protocol and the confirmation of diagnosis by MRI in all participants suggest a high internal validity. 

## Figures and Tables

**Table 1:** Demographics and baseline characteristics.

	Study Population (*n* = 30)	Peripheral Lesion (*n* = 19)	Central Lesion (*n* = 11)	*p*
Demographics				
females, *n* (%)	14 (46.7)	10 (71.4)	4 (28.6)	0.47
age (years)	55.4 ± 17.2	50.2 (15.8)	64.5 (16.2)	0.02
Cardiovascular risk profile				
diabetes mellitus type II, *n* (%)	5 (16.7)	2 (10.5)	3 (27.3)	0.33
arterial hypertension, *n* (%)	21 (70.0)	12 (63.2)	9 (81.8)	0.42
hyperlipidemia, *n* (%)	6 (20.0)	2 (10.5)	4 (36.4)	0.16
smoking, *n* (%)	8 (26.7)	6 (31.6)	2 (18.2)	0.67
coronary artery disease, *n* (%)	1 (3.3)	0	1 (9.1)	0.37
atrial fibrillation, *n* (%)	0 (0)	0	0	
sleep apnea, *n* (%)	0 (0)	0	0	
history of stroke/TIA, *n* (%)	4 (13.3)	0	4 (36.4)	0.01
Clinical symptoms				
dizziness/vertigo	100 (100)	19 (100)	11 (100)	
nausea/vomiting, *n* (%)	26 (86.7)	16 (84.2)	10 (90.9)	1.0
head motion intolerance, *n* (%)	14 (46.7)	10 (52.6)	4 (36.6)	0.47
gait unsteadiness, *n* (%)	27 (90)	16 (84.2)	11 (100)	0.28
head/neck pain, *n* (%)	3 (10.0)	2 (10.5)	1 (9.1)	1.0
double vision, *n* (%)	2 (6.7)	0	2 (18.2)	0.13
dysphagia, *n* (%)	0 (0)	0	0	
slurred speech, *n* (%)	1 (3.3)	1 (5.3)	0	1.0
paresis, *n* (%)	0 (0)	0	0	
sensory disturbance, *n* (%)	0 (0)	0	0	
new hearing impairment, *n* (%)	3 (10)	2 (10.5)	1 (9.1)	1.0
Clinical examination				
hearing loss, *n* (%)	1 (3.3)	1 (5.3)	0	1.0
gait unsteadiness, *n* (%)	26 (86.7)	15 (78.9)	11 (100)	0.27
spontaneous nystagmus, *n* (%)	17 (56.7)	12 (63.2)	5 (45.5)	0.45
smooth pursuit abnormality, *n* (%)	4 (13.3)	1 (5.3)	3 (27.3)	0.13
saccades abnormality, *n* (%)	2 (6.7)	0	1 (9.1)	0.37
gaze evoked nystagmus, *n* (%)	2 (6.7)	0	2 (18.2)	0.13
abnormal test of skew, *n* (%)	0 (0)	0	0	
ocular muscle paresis, *n* (%)	0 (0)	0	0	
abnormal conventional HIT, *n* (%)	10 (33.3)	7 (36.8)	3 (27.3)	0.7
abnormal V-HIT, *n* (%)	8 (26.7)	6 (31.6)	2 (18.2)	0.67
Caloric testing				
peripheral lesion (total), *n* (%)	15 (55.6) *	13 (72.2)	0	0.001
HINTS plus				
abnormal C-HINTS plus, *n* (%)	20 (66.7)	12 (63.2)	8 (72.7)	0.7
abnormal V-HIT assisted HINTS plus, *n* (%)	22 (73.3)	13 (68.4)	9 (81.8)	0.67
abnormal Cal-NTS plus, *n* (%)	14 (50)	6 (33.3)	9 (100)	0.001
Cranial MRI				
vertebrobasilar ischemic lesion, *n* (%)	4 (13.3)	0	4 (36.4)	0.01
Diagnosis				
vertebrobasilar stroke, *n* (%)	4 (13.3)			
posterior TIA/DWI-neg. stroke, *n* (%)	7 (23.3)			
vestibular neuritis, *n* (%)	12 (40)			
other, *n* (%)	7 (23.3)			

Data are presented for the entire study cohort as well as for subgroups of patients with and without central lesion on cMRI. *p*-values refer to comparisons between these subgroups. Data are expressed as mean ± standard deviation or percentage. Two patients with suspected posterior circulation stroke and negative initial cMRI underwent repeated cMRI (*n* = 2). * caloric testing (*n* = 27). Abbreviations: HINTS plus, head-impulse test, nystagmus type, test of skew, hearing loss; v-HIT, video-oculography assisted head-impulse test; C-HINTS plus, conventional HINTS plus; Cal-NTS plus, caloric testing combined with nystagmus testing, test of skew, and hearing loss; MRI, magnetic resonance imaging; DWI-negative stroke; clinical suspicion of stroke without confirmed lesion on diffusion weighted imaging.

**Table 2:** Accuracy of the evaluated test algorithms.

Clinical Algorithm	Sensitivity (95% CI)	Specificity (95% CI)	PPV (95% CI)	NPV (95% CI)	Accuracy (95% CI)
C-HINTS plus	72.7 (39.0–94.0)	36.8 (16.3–61.6)	40.0 (19.1–63.9)	70.0 (34.8–93.3)	50.0 (31.3–68.7)
V-HIT assisted-HINTS plus	81.8 (48.2–97.7)	31.6 (12.6–56.6)	40.9 (20.7–63.6)	75.0 (34.9–96.8)	50.0 (31.3–68.7)
Cal-NTS plus	100 (66.4–100)	66.7 (41.0–86.7)	60.0 (32.3–83.7)	100 (73.5–100)	77.8 (57.7–91.4)

Diagnostic accuracy parameters are presented with 95% confidence intervals for each evaluated test algorithm. We noted no indeterminate index test or reference standard results except for missing caloric tests from three patients, which were handled as complete case analysis. Abbreviations: C-HINTS plus, conventional HINTS plus; V-HIT, video-oculography assisted head-impulse test; Cal-NTS plus, caloric testing combined with nystagmus testing, test of skew, and hearing loss; PPV, positive predictive value; NPV, negative predictive value.

## Data Availability

All data are available from the corresponding author upon reasonable request.
